# Gene polymorphism and plasma levels of miR-155 in diabetic retinopathy

**DOI:** 10.1530/EC-19-0446

**Published:** 2019-11-11

**Authors:** E R Polina, F M Oliveira, R C Sbruzzi, D Crispim, L H Canani, K G Santos

**Affiliations:** 1 Laboratory of Human Molecular Genetics, Universidade Luterana do Brasil (ULBRA), Canoas, Brazil; 2 Endocrine Division, Hospital de Clínicas de Porto Alegre (HCPA), Porto Alegre, Brazil; 3 Department of Internal Medicine, Universidade Federal do Rio Grande do Sul (UFRGS), Porto Alegre, Brazil; 4 Cardiovascular Research Laboratory, Hospital de Clínicas de Porto Alegre (HCPA), Porto Alegre, Brazil

**Keywords:** type 2 diabetes, diabetic retinopathy, miR-155, polymorphism, gene expression, plasma

## Abstract

Circulating microRNA-155 (miR-155) is associated with type 2 diabetes mellitus (T2DM) and the rs767649 polymorphism in the *pre-MIR155* gene is associated with miR-155 expression. However, their relationship with diabetic retinopathy (DR) is still unknown. Therefore, the aim of this case-control study was to test the hypothesis that the rs767649 polymorphism in the *pre-MIR155* gene is associated with DR in South Brazilians with T2DM. We also evaluated the association of plasma levels of miR-155 with DR and the rs767649 polymorphism in a subgroup of subjects. The rs767649 polymorphism was genotyped in 139 blood donors and 546 T2DM patients (244 had no DR, 161 had non-proliferative DR and 141 had proliferative DR). miR-155 expression was quantified in 20 blood donors and 60 T2DM patients (20 from each group). Among T2DM patients, the carriership of the A allele and the A allele were more frequent in subjects with DR than in those without it (*P* < 0.05), and the A allele was independently associated with an increased risk of DR (adjusted OR = 2.12, 95% CI = 1.12–4.01). The plasma levels of miR-155 were lower in T2DM patients than in blood donors (*P* < 0.001). However, the miR-155 levels did not differ according to the presence and severity of DR or according to rs767649 genotypes among T2DM patients. These findings support that the rs767649 polymorphism in the *pre-MIR155* gene is associated with DR in T2DM and that the miR-155 plasma levels might be associated with T2DM. Additional studies are needed to further investigate their clinical significance in DR and T2DM.

## Introduction

Diabetic retinopathy (DR) is a neurodegenerative complication of diabetes (1) consistently associated with other diabetic complications and with an overall worse prognosis (2). Hyperglycaemia triggers the activation of biochemical pathways that induce inflammation and oxidative stress, thus leading to blood-retinal barrier breakdown, pericyte loss, neuronal death and angiogenesis (3).

miRNAs are small ncRNA molecules that regulate gene expression mainly through the induction of mRNA degradation and translational inhibition of their target mRNAs (4). miRNAs are key regulators of inflammatory responses associated with both early and late stages of DR (5) and their differential expression has been reported in cultured retinal cells, animal models of DR and patients with DR. The high stability of circulating miRNAs has highlighted their potential as non-invasive biomarkers of DR (6). In addition, sequence variation in genes encoding miRNAs could alter their biogenesis, maturation or target binding, thereby affecting disease susceptibility and progression (7, 8). However, to date, there are almost no studies on the association of polymorphisms in miRNA genes with DR (9, 10).

miR-155 controls both the innate and adaptive immune systems. It is expressed in several immune cell types, where it acts a positive modulator of cell proliferation, cell differentiation and effector responses, such as cytokine and antibody production (11). miR-155 is responsive to stress, oxygen (12) and many inflammatory stimuli, such as those induced by cytokines (11). In relation to glucose metabolism and insulin signalling, miR-155 was shown to be a positive regulator of insulin sensitivity and required for normal blood glucose homeostasis (13). miR-155 is also expressed in normal murine retina and retinal endothelial cells (14, 15). In retina, miR-155 is highly expressed at early embryonic stages as part of a mechanism that controls the cell fate and timing of the generation of retinal bipolar cells (16). In addition, miR-155 was suggested to modulate retinal vessel growth and remodelling during postnatal development and under ischaemia (17).

Dysregulated expression of miR-155 has been associated with pathological conditions characterized by chronic inflammation (11), neurodegeneration and neovascularization (12). miR-155 expression increases during inflammatory response and miR-155 overexpression, in turn, enhances the production of inflammatory molecules (11). However, its mechanistic role in DR is still unexplored (14) and few studies have evaluated circulating levels of miR-155 in humans with DR (18, 19). miR-155 is generated from an exon of a long ncRNA encoded in the chromosome 21q21.3 (20). Some polymorphisms in the *pre-MIR155* gene were predicted to change the secondary structure of pre-miR-155 and were shown to affect the expression and function of miR-155 in mice and humans (21). The presumably functional rs767649 polymorphism upstream of the *pre-MIR155* gene was recently associated with type 1 diabetes (T1DM) (22) and type 2 diabetes (T2DM) (23). However, its possible association with DR has not yet been investigated.

Therefore, this study was designed to investigate whether the rs767649 polymorphism in the *pre-MIR155* gene is associated with DR in South Brazilians with T2DM. In a subgroup of T2DM patients, we also evaluated whether the plasma levels of miR-155 are associated with DR, the rs767649 polymorphism and the clinical variables.

## Materials and methods

### Study population and data collection

This case-control study was carried out on 546 outpatients with T2DM and 139 presumably non-diabetic blood donors. Two hundred and ninety-eight patients were enrolled between 1999 and 2010 in the endocrinology outpatient clinics of two public tertiary care hospitals in Porto Alegre, the capital of Rio Grande do Sul State in Southern Brazil (Hospital de Clínicas de Porto Alegre – HCPA and Hospital Nossa Senhora da Conceição). The other 248 patients were enrolled between 2015 and 2017 in the endocrinology outpatient clinic of HCPA. Type 2 diabetes was defined according to the criteria of American Diabetes Association (24), and the inclusion criteria for this study were age ≥30 years at the diagnosis of diabetes, no need of permanent insulin treatment during the first year after diagnosis and no previous episodes of ketoacidosis. Patients underwent a clinical evaluation consisting of physical examination and routine laboratory examinations, such as glycated haemoglobin (HbA1c), serum creatinine and lipid profile, which were determined according to standard methods as previously described in detail (25). The CKD-EPI equation was used to estimate the glomerular filtration rate (eGFR) (26) and a questionnaire was used to collect data regarding the clinical history, including age at the diagnosis of diabetes, smoking habits, use of medication and presence of comorbidities.

Diabetic retinopathy was diagnosed by ophthalmoscopy (patients enrolled until 2010) or retinal photography (patients enrolled between 2015 and 2017) with dilated pupils by staff ophthalmologists specialized in retina from each institution, who were blinded to the patients’ molecular data. Subjects who had severe cataract or any other eye condition that impairs fundus examination were not included in the study. Retinopathy was graded according to the worst affected eye and was classified as absent (no abnormalities), non-proliferative (NPDR; microaneurysms, intraretinal haemorrhages, venous beading and intraretinal microvascular abnormalities) or proliferative (PDR; neovascularization or vitreous/preretinal haemorrhage) (27). Patients who had been previously treated with panretinal photocoagulation were also considered as having PDR. Patients with DR were defined as case subjects (*n* = 302) and patients without DR with a known diabetes duration of at least 5 years were defined as control subjects (*n* = 244). Among the 302 case subjects, 161 had NPDR and 141 had PDR.

In order to determine the frequency of the rs767649 polymorphism and the plasma levels of miR-155 in the general population, we also included 139 unrelated blood donors from the Haemotherapy Division of HCPA, who were enrolled between 2000 and 2001 (*n* = 67) or 2017 and 2018 (*n* = 72). A questionnaire was used to collect data regarding age, gender, skin colour/ethnicity, use of medication and diabetes-related information. Blood donors with a known personal and/or first-degree family history of diabetes were not included in the study, and no additional data were collected from them.

This study was approved by the Research Ethics Committees of HCPA and ULBRA (CAAE numbers 35065914.9.0000.5327 and 35065914.9.3001.5349, respectively), and all subjects gave written informed consent. Skin colour/ethnicity was self-declared and classified as white or non-white (pardo or black).

### Blood sample collection for molecular analyses

Approximately 10 mL of venous blood were drawn from each subject for molecular analyses. Until 2010, samples were collected and stored without processing at −20°C until DNA isolation. Samples collected between 2015 and 2018 were centrifuged for 15 min at 1000 ***g*** at 4°C within 3 h from collection for the separation of plasma and blood cells. Plasma samples were then aliquoted and stored at −70°C until RNA isolation and the cellular component was kept at −20°C until DNA isolation. In this study, we used the DNA samples of the 546 T2DM patients and 139 blood donors for the genotyping of the rs767649 polymorphism and RNA samples of 60 T2DM patients (20 without DR, 20 with NPDR and 20 with PDR) and 20 blood donors for the quantification of the plasma levels of miR-155.

### DNA isolation and genotyping

DNA was isolated from peripheral white blood cells by a standard salting out method (28). Genotyping of the rs767649 polymorphism in the *pre-MIR155* gene was done by real-time PCR using a pre-designed assay containing specific primers and hydrolysis probes (TaqMan® Genotyping Assay, assay ID: C_2212229_10; Thermo Fisher Scientific). Amplification reactions were done in a 7 μL total reaction volume containing 20 ng of genomic DNA, TaqMan Genotyping Master Mix (1×) (Thermo Fisher Scientific) and genotyping assay (1×). Reactions were loaded into a real-time PCR thermal cycler (StepOnePlus Real-Time PCR System; Thermo Fisher Scientific) and heated under the usual conditions specified by the manufacturer.

To ensure the accuracy of the genotyping data, a sample of each genotype was used in all PCR runs, the investigator who performed the genotyping was blinded to the patients’ clinical condition and DR status and the genotypes were determined independently by two investigators. Seventy of the 685 samples (10.2%) were randomly selected to be re-genotyped. Five samples did not amplify and the remaining had a concordance rate of 100%.

### miRNA isolation and quantification

miRNAs were isolated from 495 μL of plasma using the mirVana PARIS kit with enrichment for small RNAs (Ambion; Thermo Fisher Scientific). After protein denaturation, plasma samples were spiked-in with 50 pM of a synthetic miRNA from *Caenorhabditis elegans* (cel-miR-39; Qiagen) to control for variations during the isolation and quantification procedures.

Plasma levels of miR-155 were quantified by RT-quantitative PCR (RT-qPCR) in two separate reactions. First, RNA was reverse transcribed into cDNA and then cDNA was amplified by qPCR. RT reactions were done in a 15 μL total reaction volume containing 10 ng of the isolated RNA using a commercial kit according to the manufacturer’s protocol (TaqMan® MicroRNA RT; Thermo Fisher Scientific). Reactions were incubated in a standard thermal cycler (PTC-150; MJ Research, Waltham, USA) for 30 min at 16°C, 30 min at 42°C and 5 min at 85°C. Then, 2 μL of the cDNA reaction mixture were amplified in duplicate reactions using pre-designed miRNA assays, containing specific primers and probes (Thermo Fisher Scientific) for cel-miR-39-3p (ID number 000200) and hsa-miR-155-5p (ID number 002623). In addition to the cDNA, the 15 μL PCR reactions contained 0.75 μL of the assay, 7.5 μL of universal PCR master mix (no UNG) and 4.75 μL of nuclease-free water. Amplification reactions were incubated for 10 min at 95°C, followed by 45 cycles of 15 s at 95°C and 1 min at 60°C on StepOnePlus Real-Time PCR System (Thermo Fisher Scientific). Raw Cq values >40 were considered as undetectable.

The comparative method (2^−∆∆^^Cq^) (29) was used to determine the plasma levels of miR-155 in T2DM patients and blood donors, considering the spiked-in cel-miR-39 as the reference gene and a pool of cDNA samples as the reference sample (calibrator). The pool samples were obtained from nine subjects with T2DM randomly selected from each group (four without DR, three with NPDR and two with PDR). For statistical analysis, fold-change values were log2-transformed.

### Statistical analysis

Categorical data are shown as absolute frequency (percentage), percentage or relative frequency, and continuous variables are expressed as mean ± s.d. or median (25th and 75th percentiles). Data normality was assessed using the Shapiro–Wilk test. Categorical data were compared between groups of subjects by χ^2^-square test, followed by Bonferroni correction for multiple pairwise comparisons where appropriate or Fisher’s exact test. The χ^2^ test was also used to test for deviations from the Hardy–Weinberg equilibrium. Continuous data were compared by independent Student *t*-test, Mann–Whitney *U*, one-way ANOVA or Kruskal–Wallis, followed by the Tukey or Dunn* post hoc* analysis, as appropriate. Correlation between miR-155 and clinical variables was evaluated using the Pearson (r) or the Spearman (r_s_) correlation coefficient, as indicated by the normality test. Association of the rs767649 polymorphism with DR was evaluated by logistic regression analysis. Considering the very low frequency of the AA genotype, we tested only the dominant model for the A allele (AA + TA vs TT). Statistical analyses were done using SPSS, version 18.0 (SPSS Inc.) and WinPEPI version 11.50 (30) statistical packages. Two-tailed *P* values <0.05 were considered as statistically significant. The genotyping and expression data generated during this study are available in Supplementary Table 1 (see section on supplementary data given at the end of this article) and in a public repository (https://doi.org/10.6084/m9.figshare.9789260).

As DR was the primary outcome of our study and no previous study has investigated the association of the rs767649 polymorphism with this complication, sample size and study power were not determined *a priori*. In relation to the plasma levels of miR-155, we used the results obtained in a Chinese study (19) for the sample size calculation. The number of samples required to detect a minimum difference of 0.5 in the levels of miR-155 with a statistical power of 90% at a significance level of 0.05 was 12 (6 cases and 6 controls). Sample size was estimated using the WinPEPI statistical software.

## Results

### Characteristics of study subjects

T2DM patients were predominantly older, white and female (Table 1). As expected, most blood donors were white (69%), male (60%) and younger than T2DM subjects (mean age of 44 ± 8 years, ranging from 30 to 69 years; *P* < 0.010 for the comparison of these three variables between patients and blood donors). Subjects with PDR were older and more often male, had diabetes for longer, were more often insulin users, had lower BMI and lower eGFR as compared with those without DR. Except for the diabetes duration and use of insulin, patients with NPDR had a similar profile to those without DR (Table 1).

**Table 1:** Clinical and demographic characteristics of T2DM patients with and without DR.

Characteristic	All patients (*n* = 546)	Without DR (*n* = 244)	NPDR (*n* = 161)	PDR (*n* = 141)	*P* value
Age (years)	60.9 ± 9.0	60.1 ± 9.3^a^	60.8 ± 9.0^a,b^	62.4 ± 8.3^b^	0.050
Male gender, *n* (%)	251 (46.0)	93 (38.1)^a^	74 (46.0)^a,b^	84 (59.6)^b^	<0.001
Non-white, *n* (%)	77 (14.1)	36 (14.8)	20 (12.4)	21 (14.9)	0.766
Diabetes duration (years)	14.7 ± 7.9	13.1 ± 7.0^a^	15.0 ± 8.1^b^	17.1 ± 8.6^b^	<0.001
Insulin use (%)	54.4	38.9^a^	64.6^b^	70.2^b^	<0.001
HbA1c (%)	7.6 ± 1.9	7.6 ± 2.0^a,b^	8.0 ± 2.0^a^	7.3 ± 1.7^b^	0.018
Body mass index (kg/m^2^)	30.1 ± 5.6	31.0 ± 6.3^a^	29.8 ± 4.9^a,b^	28.6 ± 4.5^b^	<0.001
History of smoking (%)	44.9	50.0	41.0	41.1	0.173
Hypertension (%)	77.5	78.3	79.5	73.8	0.453
Systolic BP (mmHg)	141 ± 23	140 ± 22	143 ± 23	142 ± 24	0.741
Diastolic BP (mmHg)	83 ± 13	84 ± 14	82 ± 11	83 ± 12	0.702
eGFR (mL/min/1.73 m^2^)	92 (73–103)	95 (81–104)^a^	94 (79–103)^a^	76 (42–98)^b^	<0.001
Total cholesterol (mmol/L)	5.1 ± 1.4	5.0 ± 1.2	5.2 ± 1.6	5.3 ± 1.3	0.123
HDL cholesterol (mmol/L)	1.14 ± 0.32	1.16 ± 0.30	1.12 ± 0.35	1.11 ± 0.33	0.098
LDL cholesterol (mmol/L)	3.0 ± 1.1	2.9 ± 1.0	3.0 ± 1.1	3.2 ± 1.2	0.189

Data are expressed as mean ± s.d., median (25th–75th percentiles), number (percentage) or percentage. Pairwise comparisons with statistically significant differences after correction for multiple testing are indicated with the following terminology: means, medians or percentages indicated with the same letter do not differ significantly at alpha <0.05, and means, medians or percentages indicated with different letters are significantly different.

### rs767649 polymorphism in blood donors and T2DM patients with and without DR

Genotype frequencies were in agreement with those predicted by the Hardy–Weinberg equation in blood donors and T2DM patients. As shown in Table 2, genotype and allele frequencies were similar in blood donors and T2DM. In relation to DR, however, the frequency of the carriers of the A allele as well as the A allele tended to increase according to the presence and severity of this complication (Table 2). In fact, the carriership of the A allele was more frequent in patients with DR (NPDR + PDR) than in those without it (17.9 vs 9.4%, respectively; *P* = 0.007). Similarly, the A allele was more frequent in patients with DR as compared to those without this complication (8.9 vs 5.1%, respectively; *P* = 0.021).

**Table 2:** Genotype and allele frequencies of the rs767649 polymorphism in the *pre-MIR155* gene in blood donors and T2DM patients with and without DR.

Polymorphism	Blood donors (*n* = 139)	All patients (*n* = 546)	*P* value	Without DR (*n* = 244)	NPDR (*n* = 161)	PDR (*n* = 141)	*P* value
Genotype							
TT	124 (89.2)	469 (85.9)	0.441	221 (90.6)	135 (83.9)	113 (80.1)	0.008
TA	14 (10.1)	75 (13.7)		21 (8.6)^a^	26 (16.1)^a,b^	28 (19.9)^b^	
AA	1 (0.7)	2 (0.4)		2 (0.8)	0 (0.0)	0 (0.0)	
Allele							
T	0.94	0.93	0.463	0.95	0.92	0.90	0.036
A	0.06	0.07		0.05^a^	0.08^a,b^	0.10^b^	

Data are expressed as number (percentage) or relative frequency. Pairwise comparisons with statistically significant differences after correction for multiple testing are indicated with the following terminology: percentages indicated with the same letter do not differ significantly at alpha <0.05, and percentages indicated with different letters are significantly different.

Under the dominant model, the A allele was associated with an increased risk of DR (odds ratio (OR) = 2.09; 95% confidence interval (CI) = 1.24–3.52; *P* = 0.005). After controlling for the other variables that were also associated with this complication in the univariate analyses, namely, male gender (yes/no), diabetes duration (years), smoking history (positive/negative), BMI (kg/m^2^), insulin use (yes/no), eGFR (mL/min/1.73 m^2^) and total cholesterol (mmol/L), the A allele remained significantly associated with DR in the final model (Table 3).

**Table 3:** Multiple logistic regression (final model) for the association of the rs767649 polymorphism in the *pre-MIR155* gene with DR.

Variable	Adjusted OR (95% CI)	*P* value
AA + TA genotypes (vs TT genotype)	2.12 (1.12–4.01)	0.020
Body mass index (kg/m^2^)	0.94 (0.90–0.98)	0.004
Insulin use (yes/no)	3.78 (2.42–5.91)	<0.001
eGFR (mL/min/1.73 m^2^)	0.99 (0.98–1.00)	0.007
Total cholesterol (mmol/L)	1.22 (1.03–1.44)	0.024

### Plasma levels of miR-155 in blood donors and T2DM patients with and without DR

**Figure 1:**
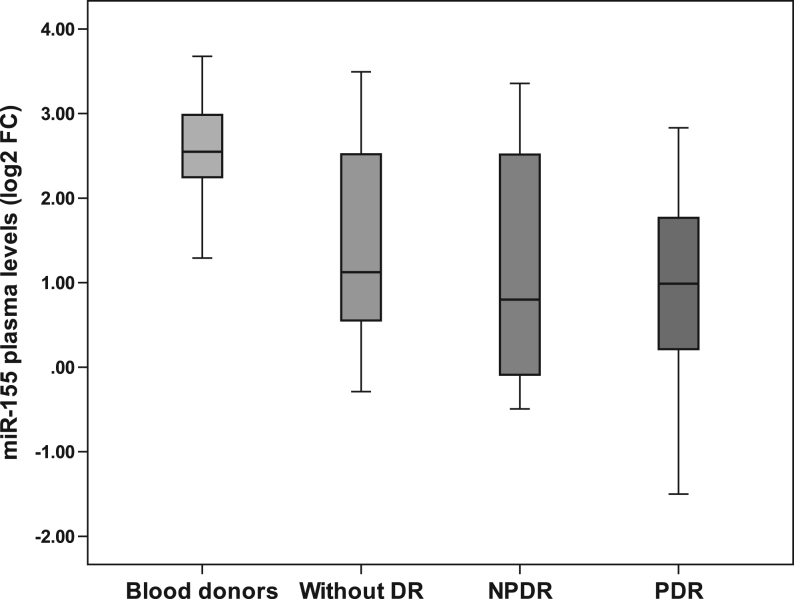
Comparative analysis of plasma levels of miR-155 in blood donors and T2DM patients with and without DR. Expression levels are expressed as log2 fold-change. Corrected *P* values <0.010 for the pairwise comparisons between blood donors and all groups of T2DM patients (without DR, NPDR and PDR).

To evaluate the plasma levels of miR-155, 20 samples were randomly selected from each group of T2DM patients. Patients had a mean age of 63 ± 8 years (ranging from 43 to 84 years), a mean diabetes duration of 18 ± 8 years (ranging from 5 to 39 years) and a mean HbA1c of 8.4 ± 1.5% (ranging from 5.5 to 11.3%). They were predominantly female (60%) and white (70%). Although blood donors were not individually matched to T2DM patients, we selected a subgroup of 20 blood donors having the same proportion of females (55%) and whites (70%) as the patient group to quantify the levels of miR-155. Indeed, the plasma levels of miR-155 were lower in T2DM patients than in blood donors, but they did not differ according to the presence or severity of DR among T2DM patients (Fig. 1 and Table 4).

**Table 4:** Plasma levels of miR-155 in blood donors and T2DM patients with and without DR.

Subject groups	miR-155 (Log2 FC)
Blood donors (*n* = 20)	2.55 (2.24–3.00)
All patients (*n* = 60)	0.99 (0.19–2.29)
*P* value^a^	<0.001
Without DR (*n* = 20)	1.12 (0.51–2.62)
NPDR (*n* = 20)	0.80 (−0.15 to 2.59)
PDR (*n* = 20)	0.99 (0.19–1.92)
*P* value^b^	0.509

Data are expressed as median (25th–75th percentiles).

^a^Comparing blood donors with all patients. ^b^Comparing patients without DR, NPDR and PDR.

FC, fold-change.

### Correlation of plasma levels of miR-155 with the rs767649 polymorphism and clinical characteristics

Considering that all but one of the 20 blood donors who had the plasma levels of miR-155 quantified were homozygous for the T allele of the rs767649 polymorphism, we could only verify whether there was an association between this gene variant and the expression of miR-155 in T2DM patients. The plasma levels of miR-155 were not different in subjects with the TT genotype (*n* = 42) as compared to those carrying the A allele (*n* = 18) (1.06 (0.15–2.23) vs 0.82 (0.41–2.46), respectively; *P* = 0.995). In addition, few correlations were found between the plasma levels of miR-155 and clinical and demographic characteristics in T2DM patients. Specifically, the median levels of miR-155 were two-fold higher in non-whites (*n* = 18) than in whites (*n* = 42) (1.66 (0.81–2.61) vs 0.82 (0.11–2.08), respectively; *P* = 0.019), and the miR-155 was weakly correlated with diastolic blood pressure (BP) (r_s_ = −0.27; *P* = 0.036).

## Discussion

In this study, we detected an independent association of the A allele of the rs767649 polymorphism in the *pre-MIR155* gene with DR in T2DM outpatients from two tertiary hospitals in Southern Brazil. We also reported that blood donors had higher plasma levels of miR-155 than T2DM patients, and among diabetic patients, the miR-155 levels were higher in non-whites than in whites. However, miR-155 expression did not vary according to the presence or severity of DR or according to rs767649 genotypes.

The frequency of the minor A allele of the rs767649 polymorphism was quite similar to that previously reported in our population (22), and the genotype and allele frequencies were almost the same in blood donors and T2DM patients. These findings contrast with those reported in studies from Brazil (22) and Italy (23), in which the A allele was associated with a lower risk of T1DM and T2DM, respectively. However, this discrepancy is not unexpected, because our study was not designed to investigate the association of the rs767649 polymorphism with T2DM and 20% of the blood donors in our population have HbA1c levels compatible with prediabetes or diabetes. This could have led to the similar distribution frequency of this polymorphism in blood donors and T2DM patients. In relation to DR, to the best of our knowledge, no study has investigated the association between the rs767649 polymorphism and this diabetic complication. We found that the A allele was associated with a two-fold increased risk of DR in T2DM patients even after controlling for clinical covariates.

Our results regarding the expression of miR-155 are in accordance with those reported in populations from Mexico (31), Egypt (32), Iran (33), Germany (34) and China (13), in which the circulating levels of miR-155 were downregulated in subjects with T2DM in comparison to healthy controls. In a streptozotocin (STZ)-nicotinamide-induced rat model of T2DM, the miR-155 expression was lower in PBMC, kidney, heart, aorta and sciatic nerve obtained from diabetic rats than in their non-diabetic counterparts (35). In genetically modified mice, the overexpression of miR-155 resulted in hypoglycaemia, improved glucose tolerance and enhanced insulin sensitivity in peripheral tissues, which were caused, at least in part, by enhanced glucose uptake and enhanced glycolysis, whereas the deficiency of miR-155 led to opposite effects (13).

In relation to DR, our results are in agreement with an Italian study in T1DM, in which miR-155-5p isolated from plasma extracellular vesicles was markedly downregulated in patients with PDR in comparison to healthy subjects (18). However, these results are opposite to those reported in a Chinese study that found higher PBMC levels of miR-155 in T2DM patients with PDR and NPDR in comparison to those without DR and healthy controls (19). These contrasting findings might be due, at least in part, to the ethnicity, patient’s profile and methodological differences in the quantification of miR-155, including the RNA isolation, qPCR and estimation of gene expression (12). Experimental studies on murine models showed that miR-155 regulates gene expression of its several targets in a biological context- and cell-type-dependent manner (36), exerting both pro- and anti-inflammatory effects (37), and may have dual effects on adaptive neovascularization (38). Given the lack of experimental evidence on the effects of miR-155 on retinal function in diabetes, it is hard to define what would be expected in relation to its expression in DR.

Previous studies have investigated the effect of miR-155 on retinal apoptosis and angiogenesis predominantly in cell culture and murine models that resemble retinopathy of prematurity (ROP) or age-related macular degeneration (AMD) (15, 17, 39, 40, 41). In this scenario, overexpression of miR-155 in the retina seemed to be detrimental (15, 17, 39). In contrast, the levels of endogenous miR-155 were decreased in retinal pigment epithelium/choroidal tissues after laser photocoagulation in mice. In this model, the intravitreous injection of miR-155 mimics markedly reduced the expression of vascular endothelial growth factor, subretinal leakage and choroidal neovascularization (40). Interestingly, an Italian study showed that the retinal and circulating levels of miRNAs could vary in the same model. miR-155 was upregulated in the retina of amyloid beta injected rats, whereas it was downregulated in serum of both AMD patients and rats (41).

Recently, a bionformatic analysis of visual perception-related genes using transcriptomic data obtained from retina samples from two mouse models of diabetes after 3 months of hyperglycaemia suggested that the expression of miR-155-5p should be downregulated in DR (42). This proposition was based on the competing endogenous RNAs (ceRNAs) hypothesis, according to which multiple miRNA targets compete with each other to bind the miRNA (43). Thus, we speculate that the downregulation of miR-155 may be detrimental in retina and this might result in even less miR-155 circulating in plasma, which could explain, at least in part, the decreased levels of miR-155 in diabetic patients in our study.

Apart from this, we have not found any difference in the plasma levels of miR-155 between T2DM patients carrying the A allele and those homozygous for the T allele. The few studies that evaluated the functionality of the rs767649 polymorphism found opposite effects on the miR-155 transcriptional activity, depending on the cell line utilized (44, 45, 46). Due to the lack of functional assays of the rs767649 polymorphism in retinal cells, its potential effect on DR is still unknown. Thus, in the absence of consistent experimental evidence regarding the functionality of this polymorphism, it is hard to explain how the A allele was associated with DR in our population, whereas the plasma levels of miR-155 were not related to it. One plausible explanation is that the rs767649 polymorphism might be in linkage disequilibrium with an adjacent region within or nearby the *pre-MIR155* that harbours the true causal variant. Another possible reason is that the plasma levels measured at one time point may not reflect their levels at the onset of DR.

Finally, our findings should be considered in light of some study limitations. First, its case-control design does not allow to establish a cause-and-effect relationship of the plasma levels of miR-155 with DR and T2DM. Second, eye fundus was examined using ophthalmoscopy in approximately half of the patients. As this method has reduced sensitivity to grade lower levels of DR (47), some subjects with mild NPDR might have been included in the group without DR. However, this would lead toward negative results. In spite of the limitations, our findings are novel as this is the first study, to our knowledge, to evaluate and report that a putatively functional polymorphism in the *pre-MIR155* gene is associated with DR. They also corroborate previous reports that circulating levels of miR-155 are downregulated in T2DM, supporting the need of additional studies to better understand the relationship of miR-155 with DR and T2DM and to evaluate whether it could have clinical value in T2DM patients.

In conclusion, the A allele of the rs767649 polymorphism in the *pre-MIR155* gene was independently associated with DR in T2DM outpatients from a South Brazilian population. The plasma levels of miR-155 were higher in blood donors than in T2DM patients, but they were not associated with DR. Moreover, the rs767649 polymorphism was not associated with the plasma levels of miR-155. Taken together, our findings provide suggestive evidence that miR-155 may be involved in the pathogenesis of DR and/or T2DM and should be investigated further to evaluate its usefulness as biomarker of these conditions.

## Supplementary Material

Supplemental table 1

## Declaration of interest

The authors declare that there is no conflict of interest that could be perceived as prejudicing the impartiality of the research reported.

## Funding

This work was supported by Fundação de Amparo à Pesquisa do Estado do Rio Grande do Sul (FAPERGS), Porto Alegre, Brazil (grant PqG 1994-2551/13-4 to K G S), Fundo de Incentivo à Pesquisa e Eventos do Hospital de Clínicas de Porto Alegre (FIPE-HCPA), Porto Alegre, Brazil (grant number 14-0645 to D C) and the Coordenação de Aperfeiçoamento de Pessoal de Nível Superior – Brasil (CAPES) – Finance Code 001 (E R P was a recipient of a postdoctoral scholarship and R C S is currently a recipient of a scholarship from this agency).
